# Postpartum depression and postpartum post-traumatic stress disorder: prevalence and associated factors

**DOI:** 10.1186/s12888-021-03432-7

**Published:** 2021-10-05

**Authors:** Ying Liu, Lan Zhang, Nafei Guo, Hui Jiang

**Affiliations:** Nursing Department, Shanghai First Maternity and Infant Hospital, Tongji University School of Medicine, No.2699, West Gaoke Road, Pudong New Area, Shanghai, 201204 China

**Keywords:** Postpartum depression, Post-traumatic stress disorder, Postpartum period, Childbirth, Mental health, China

## Abstract

**Background:**

Despite the increased global interest from researchers in postpartum depression (PPD) and postpartum post-traumatic stress disorder (PP-PTSD), studies of PPD in China have shown a wide range of variability. Indeed, the prevalence and risk factors for PP-PTSD have received little attention in China.

**Aim:**

To determine the prevalence of PPD and PP-PTSD in China, and to examine the relationships between a range of sociodemographic, pregnancy-related, and newborn-related variables, and PPD and PP-PTSD.

**Methods:**

A cross-sectional study involving 1136 women who returned to the obstetrics clinic for routine postpartum examination were enrolled. The sociodemographic, pregnancy-related, and newborn-related characteristics were collected. Social support, and PPD and PP-PTSD symptoms were measured by the Perceived Social Support Scale (PSSS), the Edinburgh Postnatal Depression Scale (EPDS), and the Perinatal Post-traumatic Stress Questionnaire (PPQ).

**Results:**

The prevalence rates of PPD and PP-PTSD symptoms were 23.5 and 6.1%, respectively. A multivariate model showed that the presence of PP-PTSD was the strongest risk factor for PPD symptoms and vice versa. Other risk factors for PPD included low sleep quality, low social support and newborn’s incubator admission. In terms of PP-PTSD symptoms, risk factors included the presence of PPD symptoms, non-Han ethnicity, and low social support, while having one child was a protective factor.

**Conclusions:**

This study addressed some gaps in the literature and provided a better understanding of PPD and PP-PTSD in China, which may contribute to early detection and intervention. Attention should be paid to women who are most susceptible to PPD and/or PP-PTSD, including those with low social support, low sleep quality, newborn’s incubator admission, non-Han ethnicity, and women with siblings.

## Introduction

The postpartum period, also known as the fourth trimester, is a time of psychological vulnerability for women. Postpartum depression (PPD) and postpartum post-traumatic stress disorder (PP-PTSD) are two common mental disorders that occurred during this period [1, 2]. PPD is defined as “a major depressive episode with peripartum onset and onset of mood symptoms occurs during pregnancy or within 4 weeks following delivery” according to the Diagnostic and Statistical Manual of Mental Disorder (DSM-5) [3, 4]. The prevalence of PPD is estimated to be 10–20% globally and higher in low-income regions (18.7, 95% CI 17.8–19.7, in low and middle income countries vs. 9.5, 95% CI 8.9–10.1, in high income countries) [5–8].

In addition to PPD, postpartum post-traumatic stress disorder (PP-PTSD), a more severe mental disorder, has gained increased attention from researchers and clinicians worldwide [2]. High co-morbid rates of PPD and PP-PTSD have been reported by some researchers (90.4% of women with PP-PTSD had also experienced PPD and 31.5% women with PPD had also experienced PP-PTSD [2, 9]. PP-PTSD is an anxiety disorder that develops as a direct consequence of a difficult or traumatic birth. Women may even suffer from PP-PTSD following a successful birth [10]. Like post-traumatic stress disorder (PTSD), the three core symptoms for PP-PTSD are re-experiencing, avoidance and numbing, and hyperarousal [10]. Although the prevalence of PP-PTSD varies widely across studies and countries, the estimated prevalence of PP-PTSD is 5.0–8.0% of women in a community sample 1–3 months postpartum, and the prevalence of clinically-significant symptoms in parturients with PP-PTSD ranged between 9.6 and 27.3% [10].

An abundance of evidence has shown that the health of a baby is directly linked to the health of the mother in the postpartum period [5, 11–13]. The association of PPD and/or PP-PTSD with poor maternal and child outcomes is well-documented [14–18], and includes impaired mother-infant bonding, low breastfeeding rates, an adverse effect on child development, and long-term somatic and psychiatric morbidity [15, 18]. A prospective impact of PPD symptoms among mothers on the social-emotional development of offspring has been reported, even 18 years after birth [19].

Despite the increased interest from researchers in PPD and PP-PTSD globally, related studies are limited in China. The prevalence of PPD has only been reported in several regions of China and there is wide variability among the studies (6.7% in Hunan [20], 11.8% in Guangzhou [21], 21.4% in Fujian [22], and 23.2% in Shanghai [23]). Moreover, there is less or no available data on PP-PTSD at present. PPD and PP-PTSD co-morbidity has been reported [2, 24], but no studies discuss the two clinical entities together in China.

The literature on risk factors for PPD, including low social support [25], low social economic status [26], cigarette smoking [27], low sleep quality [28, 29], unplanned pregnancy [26], and intimate partner violence [30] is substantial. The potential risk factors for PP-PTSD include childhood sexual abuse [31], intimate partner violence [31], low social support [32], instrumental birth, and caesarean section [33, 34]. Indeed, the potential risk factors that have been identified and tested in other countries or regions have not been thoroughly investigated in China. Due to differences in cultural values, health care policies, and welfare systems [35], risk factors for PP-PTSD in the medical literature might not apply to Chinese women.

In summary, the literature is not complete in providing a comprehensive understanding of PPD and PP-PTSD in China. Thus, the aim of this study was to address these gaps in knowledge to achieve a better understanding of PPD and PP-PTSD in China. The objective was to detect the prevalence of PPD and PP-PTSD, and to examine relationships between a range of sociodemographic and obstetric variables, and PPD and PP-PTSD. Such findings will be of great importance for raising awareness and providing appropriate interventions for PPD and PP-PTSD.

## Methods

### Design

This was a cross-sectional, descriptive study.

### Setting and participant recruitment

This study was carried out between June and December 2019 in Shanghai, one of the largest cities and the economic center of China. The participants were recruited from one of the 3-A-Class specialized hospitals, which is also one of the earliest provincial and municipal maternal and child health centers in China with > 800 beds, 30 wards, and approximately 30,000 births annually (Shanghai First Maternity and Infant Hospital, 2020). Women were recruited for the study during their routine postpartum clinical visits (usually 6–8 weeks postpartum). A convenience sample was used. Eligible women were approached by the researcher and given enough time to think about participation. After informed consent was gained, the researcher guided the women to complete an anonymous electronic questionnaire in Chinese. Eligible participants were 6–8 weeks postpartum with no complications, > 18 years of age, and had a singleton, live birth. To complete the online questionnaire, women needed to be fluent in Mandarin and have access to a mobile phone. Women with a gestational age < 28 weeks or a neonate weighing < 1500 g were excluded.

### Variable and measurements

#### Sociodemographic characteristics

Sociodemographic characteristics were collected from the participants, and included questions on age (< 35 or ≥ 35 years), household registration location (Shanghai, non-Shanghai), ethnicity (Han or non-Han), education level (primary, secondary, bachelor, or master or above), employment (unemployed/housewife or employed), the only child (yes or no), cigarette smoking (yes or no), and sleep quality (low, medium, or high), and social support.

For use in a busy clinical setting in this study, a single question was adapted and modified from the Pittsburg Sleep Quality Index (PSQI) to evaluate women’s sleep quality. The question is “How did you feel about your sleep quality in the last month?” and the response options were “low, medium, and high”.

Social support was measured using the Perceived Social Support Scale (PSSS) [36]. The PSSS is a 12-item instrument to assess support from family, friends, relatives, and colleagues. Each item is rated on a 7-point Likert scale ranging from 1(strongly disagree) to 7 (strongly agree). The total score ranges from12–84, with higher scores indicating higher perceived social support. A score between 12 and 36 indicates low support, 37–60 indicates medium support, and 61–84 indicates high support. The Chinese version of PSSS has been tested by Jiang [37] and was shown to have excellent reliability, with a Cronbach’s α of 0.99. The Cronbach’s α in this study was 0.89.

#### Pregnancy-related characteristics

Pregnancy-related characteristics were collected through the electronic medical records, included parity (primiparous or multiparous), planned pregnancy (yes or no), histories of abnormal pregnancy (yes or no), mode of birth (normal vaginal birth, instrumental birth, or cesarean section). According to the the Maternal Pregnancy Risk Assessment and Management Norms released by the National Health Commission of the People’s Republic of China [38], histories of abnormal pregnancy including miscarriage ≥3 times, history of preterm birth, perinatal death, birth defects, history of ectopic pregnancy, history of trophoblastic disease, history of previous pregnancy complications and comorbidities. Instrumental birth defined by when vaccum extractor or delivery forceps were used, and cesarean section included both planned cesarean section and emergency cesarean section. An emergency cesarean section was performed when there is a situation that immediately threatens the life of the mother or fetus. In this study setting, medical indications for emergency cesarean section included hemorrhage due to placenta praevia, placental abruption, acute fetal distress, amniotic fluid embolism, umbilical cord prolapse, uterine rupture, etc.

#### Newborn-related characteristics

Newborn-related characteristics were collected from the electronic medical records, and included baby gender (boy or girl), weight of the neonate (low birthweight: 1500 g–2499 g, normal birthweight: 2500 g–3999 g, or fetal macrosomia: ≥4000 g), gestational age (preterm: 28–36^+ 6^ weeks, full term: 37–41^+ 6^ weeks, or postdates ≥42 weeks), newborn’s Apgar score in 1 min (0–3: severe asphyxia, 4–7: mild asphyxia, 8–10: no asphyxia), Apgar score in 5 min (0–3, severe asphyxia, 4–7, mild asphyxia, 8–10, no asphyxia), NICU admission (yes or no), incubator admission (yes or no), and diagnosis of hyperbilirubinemia (yes or no).

#### Psychosocial characteristics

Both PPD and PP-PTSD were collected through self-reported questionnaires.

Symptoms of PPD were measured with the Edinburgh Postnatal Depression Scale (EPDS), the most widely used tool for identifying possible PPD and has been recommended by the American College of Obstetricians and Gynecologists (ACOG) and the American Academy of Pediatrics (AAP) [3]. The EPDS is a 10-item, self-report instrument, first published by Cox et al., [39, 40] and translated into over 60 languages. Each item of EPDS is rated on 4-point range from 0 (no, not at all) to 3 (yes, most of the time), and the total score ranges from 0 to 30, with higher scores indicating greater severity of symptoms. We used the Chinese version of the EPDS, which has been validated in Chinese parturients with reliability and validity [41], and the Cronbach’s α for EPDS in the current study was 0.879. A cut-off score ≥ 13, with sensitivity (86%) and specificity (78%) [40] was used in this study.

Symptoms of PP-PTSD were measured with the Perinatal Post-traumatic Stress Questionnaire (PPQ). The PPQ is a self-rating scale, developed by DeMier [42] and based on DSM-IV to identify women suffering from PTSD symptoms at 1–18 months postpartum. The PPQ has 14 items, with each item scoring from 0 (not at all) to 4 (often for more than 1 month). The total score ranges from 0 to 56, with a score ≥ 19 indicating that PP-PTSD symptoms exist. The reliability and validity of the Chinese version of the PPQ were tested by Zhang [43] with a Cronbach’s α of 0.84; the test-retest reliability was 0.88. The Cronbach’s α in this study was 0.83.

### Procedure and ethical consideration

Ethical approval for the study was obtain from the hospital Ethics Committee. Researchers approached potential participants and gave a brief introduction of the study (research purpose, potential impact, and participants’ rights). Women were informed that their participation was entirely voluntary, and whether they agreed to participate or not did not impact their treatment. Informed consent was obtained from participants at the beginning of the online questionnaire. All participants were assured that their data would be kept confidential and only accessed by the researcher.

### Data analyses

Data were analyzed using the Statistical Package for Social Sciences (SPSS, version 22.0 for Windows). Scores of EPDS and PPQ are presented as the mean (M) and standard deviation (SD). To facilitate statistical analysis and interpretation of the results, the PSSS, EPDS, and PPQ scores were converted into categorical variables. Frequencies and percentages are presented for all categorical variables. A chi-square test and logistic regression analysis were performed to evaluate the association of factors related to PPD and PP-PTSD. Variables were included into the multivariate logistic regression model if the variables had a significant association (*P* ≤ 0.20) by the χ^2^ test or deemed important enough clinically, despite no statistical significance. All statistics were performed using two-sided tests, and the significance level was set at a *P* <  0.05.

## Results

### Characteristics of participants

During the research period, 1200 eligible women were approached, and 1136 of them agreed to participate in the study and completed the questionnaire (94.6% response rate). The characteristics of the participants are shown in Table 1. The mean age of the participants was 30.16 ± 3.91 years. The majority of participants had a bachelor’s degree or above (*n* = 961 [84.6%]), 2 participants (0.2%) reported smoking cigarettes during pregnancy, 479 participants (42.2%) had only 1 child in the family, and 821 participants (72.3%) were primiparas.

**Table 1:** Characteristics of the participants (*N* = 1136)

Variables	Total n (%)	No PPD	PPD symptoms	*P* value	No PP-PTSD	PP-PTSD symptoms	*P* value
Socio-demorgraphic characteristics							
Age				0.066			0.404
< 35 years	983 (86.5)	743 (85.5)	240 (89.9)		921 (86.3)	62 (89.9)	
≥ 35 years	153 (13.5)	126 (14.5)	27 (10.1)		146 (13.7)	7 (10.1)	
Household registration location				**0.002**			**0.005**
non-Shanghai	801 (70.5)	593 (68.2)	208 (77.9)		742 (69.5)	59 (85.5)	
Shanghai	335 (29.5)	276 (31.8)	59 (22.1)		325 (30.5)	10 (14.5)	
Ethnicity				**0.019**			**0.016**
non-Han	14 (1.2)	7 (0.8)	7 (2.6)		11 (1.0)	3 (4.3)	
Han	1122 (98.8)	862 (99.2)	260 (97.4)		1056 (99.0)	66 (95.7)	
Education level				0.300			0.067
primary education	46 (4.0)	35 (4.0)	11 (4.1)		41 (3.8)	5 (7.2)	
secondary education	129 (11.4)	98 (11.3)	31 (11.6)		116 (10.9)	13 (18.8)	
bachelor	764 (67.3)	575 (66.2)	189 (70.8)		721 (67.6)	43 (62.3)	
master or above	197 (17.3)	161 (18.5)	36 (13.5)		189 (17.7)	8 (11.6)	
Employment				0.162			0.328
unemployed / housewife	431 (37.9)	320 (36.8)	111 (41.6)		401 (37.6)	30 (43.5)	
employed	705 (62.1)	549 (63.2)	156 (58.4)		666 (62.4)	39 (56.5)	
The only child				**< 0.001**			**0.001**
no	657 (57.8)	477 (54.9)	180 (67.4)		604 (56.6)	53 (76.8)	
yes	479 (42.2)	392 (45.1)	87 (32.6)		463 (43.4)	16 (23.2)	
Cigarette smoking				> 0.999 ^a^			> 0.999 ^a^
no	1134 (99.8)	867 (99.8)	267 (100.0)		1065 (99.8)	69 (100.0)	
yes	2 (0.2)	2 (0.2)	0 (0.0)		2 (0.2)	0 (0.0)	
Sleep quality				**< 0.001**			**< 0.001**
low	78 (6.9)	42 (4.8)	36 (13.5)		64 (6.0)	14 (20.3)	
medium	634 (55.8)	466 (53.6)	168 (62.9)		597 (56.0)	37 (53.6)	
high	424 (37.3)	361 (41.5)	63 (23.6)		406 (38.1)	18 (26.1)	
Social support				**< 0.001**			**< 0.001**
low (PSSS score 12–36)	22 (1.9)	3 (0.3)	19 (7.1)		15 (1.4)	7 (10.1)	
medium (PSSS score 37–60)	357 (31.4)	203 (23.4)	154 (57.7)		312 (29.2)	45 (65.2)	
high (PSSS score 61–84)	757 (66.6)	663 (76.3)	94 (35.2)		740 (69.4)	17 (24.6)	
Pregnancy-related characteristics							
Parity				0.750			0.554
primiparous	821 (72.3)	626 (72.0)	195 (73.0)		769 (72.1)	52 (75.4)	
multiparous	315 (27.7)	243 (28.0)	72 (27.0)		298 (27.9)	17 (24.6)	
Planned pregnancy				0.069			0.071
no	276 (24.3)	200 (23.0)	76 (28.5)		253 (23.7)	23 (33.3)	
yes	860 (75.7)	669 (77.0)	191 (71.5)		814 (76.3)	46 (66.7)	
Histories of abnormal pregnancy				0.333			0.851
no	1107 (97.4)	849 (97.7)	258 (96.6)		1040 (97.5)	67 (97.1)	
yes	29 (2.6)	20 (2.3)	9 (3.4)		27 (2.5)	2 (2.9)	
Mode of birth				0.633			0.296
normal birth	709 (62.4)	540 (62.1)	169 (63.3)		670 (62.8)	39 (56.5)	
instrumental birth	15 (1.3)	13 (1.5)	2 (0.7)		15 (35.8)	0 (0.0)	
cesarean section	412 (36.3)	316 (36.4)	96 (36.0)		382 (1.4)	30 (43.5)	
Newborn-related characteristics							
Baby gender				0.854			0.496
boy	597 (52.6)	458 (52.7)	139 (52.1)		558 (52.3)	39 (56.5)	
girl	539 (47.4)	411 (47.3)	128 (47.9)		509 (47.7)	30 (43.5)	
Weight of neonate				0.812			0.556
low birthweight (1500-2499 g)	28 (2.5)	20 (2.3)	8 (3.0)		25 (2.3)	3 (4.3)	
normal birthweight (2500-3999 g)	1034 (91.0)	792 (91.1)	242 (90.6)		973 (91.2)	61 (88.4)	
fetal macrosomia (≥4000 g)	74 (6.5)	57 (6.6)	17 (6.4)		69 (6.5)	5 (7.2)	
Gestational age				0.857^a^			0.964 ^a^
preterm (28–36^+ 6^ weeks)	47 (4.1)	36 (4.1)	11 (4.1)		44 (4.1)	3 (4.3)	
full term (37–41^+ 6^ weeks)	1088 (95.8)	832 (95.7)	256 (95.9)		1022 (95.8)	66 (95.7)	
postdates (≥42 weeks)	1 (0.1)	1 (0.1)	0 (0.0)		1 (0.1)	0 (0.0)	
Apgar score in 1 min				**0.007**^**a**^			**0.033**^**a**^
0–3 (severe asphyxia)	1 (0.1)	0 (0.0)	1 (0.4)		0 (0.0)	1 (1.4)	
4–7 (mild asphyxia)	8 (0.7)	3 (0.3)	5 (1.9)		7 (0.7)	1 (1.4)	
8–10 (no asphyxia)	1127 (99.2)	866 (99.7)	261 (97.8)		1060 (99.3)	67 (97.1)	
Apgar score in 5 min				0.235^a^			0.061^a^
4–7 (mild asphyxia)	1 (0.1)	0 (0.0)	1 (0.4)		0 (0.0)	1 (1.4)	
8–10 (severe asphyxia)	1135 (99.9)	869 (100.0)	266 (99.6)		1067 (100.0)	68 (98.6)	
NICU admission				0.099			0.177
no	983 (86.5)	760 (87.5)	233 (83.5)		927 (86.9)	56 (81.2)	
yes	153 (13.5)	109 (12.5)	44 (16.5)		140 (13.1)	13 (18.8)	
Incubator admission				**0.017**			0.261^a^
no	1075 (94.6)	830 (95.5)	245 (91.8)		1012 (94.8)	63 (91.3)	
yes	61 (5.4)	39 (4.5)	22 (8.2)		55 (5.2)	6 (8.7)	
Hyperbilirubinemia				0.550			0.065^a^
no	1088 (95.8)	834 (96.0)	254 (95.1)		1025 (96.1)	63 (91.3)	
yes	48 (4.2)	35 (4.0)	13 (4.9)		42 (3.9)	6 (8.7)	
Psychosocial characteristics							
PPD symptoms							**< 0.001**
no (EPDS score < 13)	869 (76.5)				855 (80.1)	14 (20.3)	
yes (EPDS score ≥ 13)	267 (23.5)				212 (19.9)	55 (79.7)	
PP-PTSD symptoms				**< 0.001**			
no (PPQ score < 19)	1067 (93.9)	855 (98.4)	212 (79.4)				
yes (PPQ score ≥ 19)	69 (6.1)	14 (1.6)	55 (20.6)				

a: Fisher’s exact probability test.

Bold values indicate statistical significance at a *P* <  0.05.

*PPD* Postpartum depression, *PP-PTSD* Postpartum post-traumatic stress disorder, *PSSS* Perceived Social Support Scale, *EPDS* Edinburgh Postnatal Depression Scale, *PPQ* Perinatal Post-traumatic Stress Questionnaire.

### Prevalence and factors for PPD symptoms

The mean EPDS score was 9.54 ± 4.46 (range = 3–25), and 267 (23.5%) women had PPD symptoms with an EPDS score ≥ 13. Based on univariate analysis (Table 1), there was a significant difference in the prevalence of PPD symptoms among the groups with respect to the household registration location, ethnicity, one child families, sleep quality, social support, newborn’s Apgar score in 1 min, newborn’s incubator admission and PP-PTSD symptoms (*P* <  0.05) using a chi-square test.

In the multivariate model (Table 2), women with low sleep quality (OR, 3.658; 95% CI, 1.995–6.706) or medium sleep quality (OR, 1.924; 95% CI, 1.314–2.760) were more likely to have PPD symptoms than women with high sleep quality. Compared to women with a low level of social support, medium or high social support reduced the odds of PPD symptoms (OR, 0.128; 95% CI, 0.035–0.465 and OR, 0.030; 95% CI, 0.008–0.108, respectively). Women with a newborn who had incubator admission reported a higher risk of PPD symptoms than women without (OR, 2.109; 95CI, 1.117–3.982). The presence of PP-PTSD symptoms increased the risk of PPD symptoms (OR, 9.170; 95% CI, 4.773–17.617).

**Table 2:** Factors associated with PPD symptoms based on multivariate analyses (N = 1136)

Characteristics	ß	Wald	*P* value	OR (95%CI)
Sleep quality				
low	**1.292**	**17.493**	**< 0.001**	**3.640 (1.987–6.668)**
medium	**0.653**	**12.545**	**< 0.001**	**1.922 (1.339–2.760)**
high				reference
Social support				
low (PSSS score 12–36)				reference
medium (PSSS score 37–60)	**−2.101**	**10.202**	**0.001**	**0.122 (0.034–0.444)**
high (PSSS score 61–84)	**−3.535**	**28.940**	**< 0.001**	**0.029 (0.008–0.106)**
Incubator admission				
no				reference
yes	**0.746**	**5.289**	**0.021**	**2.109 (1.117–3.982)**
PP-PTSD symptoms				
no (PPQ score < 19)				reference
yes (PPQ score ≥ 19)	**2.216**	**44.243**	**< 0.001**	**9.170 (4.773–17.617)**

Bold values indicate statistical significance.

ß: standardized regression coefficients. OR: adjusted odds ratio, adjusted for household registration location, ethnicity, employment, one child family, sleep quality, social support, planned pregnancy, newborn’s Apgar score in 1 min, NICU admission, incubator admission, and PP-PTSD symptoms. 95% CI: 95% confidence interval.

*PPD* Postpartum depression, *PSSS* Perceived Social Support Scale, *PP-PTSD* Postpartum post-traumatic stress disorder, *PPQ* Perinatal Post-traumatic Stress Questionnaire.

### Prevalence and factors for PP-PTSD symptoms

The mean PPQ score was 6.30 ± 6.37 (range = 0–45), and 69 (6.1%) women had PP-PTSD symptoms with a PPQ score ≥ 19. Based on univariate analysis (Table 1), there were significant differences in the prevalence of PP-PTSD symptoms among the groups with respect to household registration location, ethnicity, one child families, sleep quality, social support, newborn’s Apgar score in 1 min, and PPD symptoms (*P* < 0.05) using a chi-square test.

In the multivariate model (Table 3), women of non-Han ethnicity were more likely to experience PP-PTSD symptoms that women of Han ethnicity. Women with pregnancy-induced hypertension had a higher risk of PP-PTSD symptoms than women who did not have pregnancy-induced hypertension (OR, 5.041; 95% CI, 1.724–14.743). Being the only child in the family and having high social support reduced the odds of PP-PTSD symptoms (OR, 0.436; 95% CI, 0.023–0.828 and OR, 0.211; 95% CI, 0.069–0.643, respectively). The presence of PPD symptoms increased the risk of PP-PTSD symptoms (OR, 9.807; 95% CI, 5.071–18.962).

**Table 3:** Factors associated with PP-PTSD symptoms based on multivariate analyses (*N* = 1136)

Characteristics	ß	Wald	*P* value	OR (95%CI)
Ethnicity				
non-han	**1.542**	**4.253**	**0.039**	**4.672 (1.079–20.217)**
Han				reference
The only child				
no				reference
yes	**−0.830**	**6.436**	**0.011**	**0.436 (0.023–0.828)**
Social support				
low (PSSS score 12–36)				reference
medium (PSSS score 37–60)	−0.487	0.859	0.354	0.614 (0.219–1.722)
high (PSSS score 61–84)	**−1.555**	**7.498**	**0.006**	**0.211 (0.069–0.643)**
PPD symptoms				
no (EPDS score < 13)				reference
yes (EPDS score ≥ 13)	**2.283**	**46.064**	**< 0.001**	**9.807 (5.072–18.962)**

Bold values indicate statistical significance.

ß: standardized regression coefficients. OR: adjusted odds ratio, adjusted for household registration location, ethnicity, education level, one child family, sleep quality, social support, planned pregnancy, newborn’s Apgar score in 1 min, Apgar score in 5 min, NICU admission, hyperbilirubinemia, and PPD symptoms. 95% CI: 95% confidence interval.

*PP-PTSD* Postpartum post-traumatic stress disorder, *PSSS* Perceived Social Support Scale, *PPD* Postpartum depression, *EPDS* Edinburgh Postnatal Depression Scale.

## Discussion

### Prevalence of PPD and PP-PTSD symptoms

Maternal mental disorders, including PPD, anxiety, and PP-PTSD, are common but often overlooked in China. Due to diversity of the regional economy, culture and policy, and heterogeneity in the prevalence of maternal health disorders has been reported among regions of China [20–22]. In our study, the prevalence of PPD symptoms was 23.5%, which was within the range of 8–26% reported in a global review article that included 58 articles (*N* = 37,294 women) [44], but higher than studies reported in other areas of China [20–22]. This finding suggests that PPD symptoms among women in Shanghai may be high in China and in need of more attention. One explanation is that this study conduct in Shanghai, one of the most economically developed regions in China, the increasing public awareness of maternal mental health, and practice of routine screening PPD may contribute to the increased detection of PPD symptoms. Another possible explanation is that Shanghai has a large amount of transient and floating population. In this study, only 29.5% of the participant are Shanghainese (with local identification). The high mobility of society may weaken the individual’s identity of the residential community and reduce the availability of social support, then results in poor mental health.

We reported a PP-PTSD symptom prevalence of 6.1% among parturients 6–8 weeks postpartum. This finding is similar to that reported by Yildiz et al. [14] (5.8%, 4–6 weeks, 1.4%, 3 months; and 6.8%, 6 months) in a review article that included 28 international studies, but less than the results reported by Radoš et al., [45] (11.8% as measured by the City Birth Trauma Scale). This inconsistency may be explained by the different measures.

It is worth noting that due culture and social norm differences, Chinese often adopt a more conservative attitude toward mental disorders and tend to conceal their psychological discomfort [24]. This fact may contribute to a lower rate of reporting mental disorders in China.

### Influencing factors

Previous studies have shown that mental health disorders often co-exist and affect each other [2, 24]. Suffering from prior depression leads to a strong vulnerability for developing PP-PTSD, while prior PTSD has been shown to render women vulnerable to PPD [2]. In this study, we also confirm that PP-PTSD is the strongest risk factor for PPD and vice versa. Dekel et al. [2] emphasized that co-existing PPD and PP-PTSD signify a distinct condition and should be deemed as an independent childbirth outcome. Biological studies of non-postpartum samples have shown that compared to depression or PTSD alone, co-morbid depression and PTSD are associated with distinctive biomarkers [46, 47].

Social support was an important protective factor for PPD and PP-PTSD in the current study, which is consistent with previous studies [24, 48]. Being a mother and taking care of a newborn in the early postpartum period can be very challenging, which involves hormonal imbalance, blood loss, pain, and sleep deprivation [10]. Even a full-term delivery with health outcomes may be associated with fear, loss of control, and body integrity [10]. All of these factors result in a greater need of support from family, friends, neighbors, colleagues, and other groups. Without adequate social support, women will be at high risk for mental disorders. Moreover, different aspects of social support are recommended in different contexts. For example, informational and instrumental support may be more in need at 3 weeks postpartum, while social integration may be more in need at 8 weeks postpartum [49].

The association between sleep quality and PPD was confirmed in our study, which is consistent with previous studies [29, 50]. Poor sleep quality has been reported in women from late pregnancy to even 3 years postpartum [50]. Thus, a flexible suite of interventions targeting poor sleep quality may be a good way to solve mental health problems [50].

Our results showed that compared to women of Han ethnicity, women of non-Han ethnicity were more likely to report PP-PTSD symptoms. China, it should be noted, is one of the world’s largest multi-ethnic countries, with 55 ethnic minorities accounting for approximately 8.5% of the overall population [51]. Compared with the Han ethnicity, the non-Han ethnicity (ethnic minorities) is often under greater social and economic pressure and has more difficulties to get advanced medical resources [51]. The health inequality in maternal and child care may contribute to the differences in maternal mental health status.

An interesting finding in the current study was that being the only child in a family was a protective factor for PP-PTSD. The majority of our participants were born under the influence of the one-child policy in China (1978–2015) and approximately one-half of the participants (*n* = 479 [42.2%]) were the only child in the family [52]. Compared to women with siblings, women who were the only child in the family usually had more parental attention, higher family support, more opportunities for a higher education, and lower economic pressures [52]. This fact may explain the protective role of the one child status and PP-PTSD symptoms. The moderating role of the one child status in direct and indirect relationships between negative life events and PTSD symptoms have been tested in non-postpartum samples [53, 54].

Several factors, such as low education [48], cigarette smoking [48], primiparity [48] and a history of a cesarean section [33, 34] have been reported to be associated with PP-PTSD in some studies, but not confirmed in our study. The reason for this discrepancy might be differences among study populations, regions, study settings, and the measurement tools used. In the study sample, the education level of women was relatively high, with 84.6% having a bachelor’s degree or above and the number of women who reported cigarette abuse was extremely low (2 of 1143). With respect to parity, the results are conflicting. In contrast to previous studies [33, 34], Abedian et al., [55] reported that multiparas had a higher rate and mean score of PP-PTSD than primiparas. This finding may be attributed to lower postpartum social support among multiparas and a higher rate of unplanned pregnancy than primiparas [55]. More quantitative research is needed to further evaluate these associations.

Furthermore, newborn-related factors, only newborn’s incubator admission had been confirmed to be related with women’s PPD symptoms in this study, other factors such as low birth weight, preterm birth, NICU admission, and diagnosis of hyperbilirubinemia showed no association with PPD or PP-PTSD in the current study, which was consistent with a study of 1531 women in Spain with PP-PTSD assessed by the PPQ [33]. A possible explanation is that women with newborn had complications may receive more meticulous care and support from both healthcare providers and family member. It can be assumed that compared with the occurrence of newborn complications itself, how the mothers treat and deal with those events may have a greater impact on her psychology. Qualitative and quantitative research can be carried out to further explore these issues. Moreover, the newborn’s Apgar score in 1 min was correlated with PPD and PP-PTSD symptoms based on univariate analysis but failed to reach statistical significance base on multivariable analysis. The limited number of newborns with mild or severe asphyxia (9/1136) in this study may lead to the contingency of the results.

### Implications

Although there has been an extensive improvement in prenatal care, psychological care is still largely overlooked. Undetected and untreated mental disorders may lead to a burden on women, their family, the health system, and whole society. Considering the incidence of disease, a routine screening of PPD for all women and PP-PTSD for women at risk during the postpartum period was recommended. Because the comorbidity of mental health disorders existed, for women who exhibited early signs and symptoms of mental health, a more comprehensive and multiple screening of mental disorders is suggested. Obstetricians and midwives can play a crucial role in the initial assessment. For women who are identified to be at risk for PPD or PP-PTSD, prompt referral to multidisciplinary specialists for further assessment and interventions is needed.

## Limitations

Firstly, the nature of a cross-sectional study has its own limitation. Further longitudinal studies are needed to test how those risk factors play a role in the development or maintenance of the symptoms, and to generate more robust and generalizable findings. Secondly, the absence of strong measures on sleep quality may limit conclusions on the accuracy or internal validity of the measure. Moreover, self-report measures (EPDS and PPQ) for possible PPD or PP-PTSD symptoms were used in this study, which may result in a higher prevalence than clinical diagnostic criteria. Reassuringly, the difference between clinical interviews and questionnaires measures was not significant, and women who suffer from PPD or PP-PTSD symptoms, but do not fulfill diagnostic criteria may also benefit from treatment [14]. Finally, some important factors such as marital problems, pregnancy depression were not collected in this study, which needs to be further confirmed by later studies.

## Conclusions

This study addressed some gaps in the literature and provided a better understanding of PPD and PP-PTSD in China, which may contribute to early detection and early intervention. In this study, the prevalence of PPD and PP-PTSD symptoms was 23.5 and 6.1% in Shanghai, China, respectively, and co-existing PPD and PP-PTSD was also confirmed in the study sample. More attention should be paid to women who are most susceptible to PPD and/or PP-PTSD, including those with low social support, low sleep quality, non-Han ethnicity, and women with siblings.

## Data Availability

Datasets used and analyzed in this study are available from the corresponding author on reasonable request.
